# Poisons and Their Antidotes

**Published:** 1884-10

**Authors:** 


### POISONS AND THEIR ANTIDOTES.

### *I.—ACIDS.*

HYDROCHLORIC ACID—MURIATIC ACID (SPIRITS OF SALT) NITRIC ACID (AQUA FORTIS)—SULPHURIC ACID (OIL OF VITRIOL).

ANTIDOTE.—An antidote should be given at once, to neutralize the acid. Strong soap-suds is an efficient remedy, and can always be obtained. It should be followed by copious draughts of warm water or flax-seed tea. Chalk, magnesia, soda or saleratus (with water) or lime-water are the best remedies. When sulphuric acid has been taken, water should be given sparingly; because, when water unites with this acid, intense heat is produced.

OXALIC ACID POISONING.—THE ANTIDOTE.

ANTIDOTE.—Oxalic acid resembles epsom salts, in appearance, and may easily be mistaken for it. The antidotes are magnesia, or chalk mixed with water.

PRUSSIC ACID—OIL OF BITTER ALMONDS—LAUREL WATER—CYANIDE OF POTASSIUM (USED IN ELECTROTYPING).

ANTIDOTE.—Cold douche to the spine. Chlorine water, or water of ammonia largely diluted, should be given, and the vapor arising from them may be inhaled.

### *II.—ALKALIES AND THEIR SALTS.*

AMMONIA (HARTSHORN) LIQUOR, OR WATER OF AMMONIA—POTASSA (CAUSTIC POTASH)—STRONG LEY—CARBONATE OF POTASSA (PEARL-ASH)—NITRATE OF POTASSA (SALTPETRE).

ANTIDOTE.—Give the vegetable acids, diluted, as weak vinegar, acetic, citric, or tartaric acid dissolved in warm water. Castor oil, linseed oil, and sweet oil, may also be used; they form soaps when mixed with the free alkalies, which they thus render harmless. The poisonous effects of saltpetre must be counteracted by taking mucilaginous drinks freely, so as to produce vomiting.

### *III.—ALCOHOL.*

BRANDY — WINE — ALL OF THE SPIRITUOUS LIQUORS.

ANTIDOTE.—Give, as an emetic, ground mustard, or tartar emetic. If the patient cannot swallow, introduce a stomach pump; pour cold water on the head.

### *IV.—GASES.*

CHLORINE — CARBONIC ACID GAS—CARBIONC OXIDE — FUMES OF BURNING CHARCOAL — SULPHURETTED HYDROGEN — ILLUMINATING OR COAL GAS.

ANTIDOTE.—For poisoning by chlorine, inhale cautiously, ammonia (hartshorn). For other gases cold water should be poured upon the head, and stimulants cautiously administered; artificial respiration.

—§—

### *V.—METALS.*

ANTIMONY—TARTAR EMETIC—WINE OF ANTIMONY — ETC.

ANTIDOTE —If vomiting has not occurred, it should be produced by tickling the throat with the finger or a feather, and the abundant use of warm water. Astringent infusions, such as common tea, oak bark and solution of tannin, act as antidotes.

—§—

### *VI.—ARSENIC.*

WHITE ARSENIC — FOWLER'S SOLUTIONS — FLY POWDER—COBALT; PARIS GREEN, ETC.

ANTIDOTE.—Produce vomiting at once, with a tablespoonful or two, of powdered mustard in a glass of warm water; or with ipecac. The antidote is hydrated proxide of iron. If Fowler's solution has been taken, lime water must be given.

—§—

### *VII.—COPPER.*

ACETATE OF COPPER (VERDIGRIS)—SULPHATE OF COPPER (BLUE VITRIOL) — FOOD COOKED IN DIRTY COPPER VESSELS, OR PICKLES MADE GREEN BY COPPER.

ANTIDOTE.—Milk or white of eggs, with mucilaginous drinks (flaxseed tea, etc., should be given freely.

—§—

### *VIII.—IRON.*

SULPHATE OF IRON (COPPERAS), ETC., ETC.

ANTIDOTE.—Carbonate of soda in some mucilaginous drink, or in water, is an excellent antidote.

—§—

### *IX.—LEAD.*

ACETATE OF LEAD (SUGAR OF LEAD)—CARBONATE OF LEAD: WHITE LEAD —WATER KEPT IN LEADEN PIPES OR VESSELS—FOOD COOKED IN VESSELS GLAZED WITH LEAD.

ANTIDOTE.—Induce vomiting with ground mustard or common salt in warm water. The antidote for soluble preparations of lead is epsom salts; for the insoluble forms, sulphuric acid largely diluted.

### *X.—MERCURY.*

BICHLORIDE OF MERCURY (CORROSIVE SUBLIMATE)—AMMONIATED MERCURY (WHITE PRECIPITATE)—RED OXIDE OF MERCURY (RED PRECIPITATE)—SULPHURET OF MERCURY VERMILION.

ANTIDOTE.—The white of eggs, or wheat flour beaten up with water and milk, are the best antidotes.

§

### *XI.—SILVER POISONING.*

NITRATE OF SILVER (LUNAR CAUSTIC).

ANTIDOTE.—Give a teaspoonfull of common salt in a tumbler of water. It decomposes the salts of silver and destroys their activity.

§

### *XII.—ZINC POISONING.*

SULPHATE OF ZINC, ETC, (WHITE VITRIOL).

ANTIDOTE.—The vomiting may be relieved by copious draughts of warm water. The antidote is carbonate of soda administered in water.

%

### *XIII.—NARCOTIC POISONS.*

OPIUM (LAUDANUM, PAREGORIC, SALTS OF MORPHIA, GODFREY'S CORDIAL, DALBY'S CARMINATIVE SOOTHING SYRUP, CHOLERA MIXTURES)—ACONITE—BELLADONNA—HEMLOCK—STRAMONIUM—DIGITALIS—TOBACCO—HYOSCIAMUS—NUX VOMICA—STRYCHNINE.

ANTIDOTE.—Evacuate the stomach by the most active emetics, as mustard, alum, or sulphate of zinc. The patient should be kept in motion, and cold water dashed on the head and shoulders. Strong coffee must be given. The physician will use the stomach pump and electricity. In poisoning by nux vomica, or strychnine, etc., chloroform or ether should be inhaled to quiet the spasms.

§

### *XIV.—IRRITANT VEGETABLE POISONS.*

CROTON OIL—OIL OF SAVINE—POKE—OIL OF TANSY, ETC.

ANTIDOTE—If vomiting has taken place, it may be rendered easier by copious draughts of warm water. But if symptoms of insensibility have come on without vomiting, it ought to be immediately excited, by ground mustard mixed with warm water, or some other active emetic; and after its operation, an active purgative should be given. After evacuating as much as possible of the poison, strong coffee, or vinegar and water, may be given with advantage.

### *XV.—POISONOUS FISH.*

### CONGER EEL — MUSSELS — CRABS, ETC.

**ANTIDOTE.**—Evacuate, as soon as possible, the contents of the stomach and bowels, by emetics (ground mustard mixed with warm water or powdered alum), and castor oil; drinking freely, at the same time, of vinegar and water. Ether, with a few drops of laudanum, mixed with sugar and water, may afterward be taken freely.

——§——

### *XVI.—POISONOUS SERPENTS.*

**ANTIDOTE.**—A ligature or handkerchief should be applied moderately tight above the bite, and a cupping-glass over the wound. The patient should drink freely of alcoholic stimulants containing a small quantity of ammonia. The physician may inject ammonia into the veins.

——%——

### *XVII.--POISONOUS INSECTS.*

### STINGS OF SCORPION, HORNET, WASP, BEE, ETC.

**ANTIDOTE.**—A piece of rag moistened with a solution of carbolic acid, may be kept on the affected part until the pain is relieved, and a few drops of carbolic acid may be given frequently in a bottle of water. The sting may be removed—according to "Hutchison's Phisiology"—by making strong pressure around it with the barrel of a small watch key.

---

### INSANITY IN FRANCE.

One of the most striking features of modern French life is the rapid increase of insanity, the number of cases of which, and especially those induced by alcoholism, is becoming larger each year. In 1882 there were 13,434 admissions into the asylums, of which 10,181 were new cases, the total number under treatment in the year being 58,760, of which 27,000 were men, and 31,000 women, showing that females are the most liable to the disease. That they are less influenced by treatment is shown by the fact that the average duration of treatment for men is 276 days, while for women it is 295 days. In France there are 61 public and 42 private asylums, of which 9 are for men only, and 14 for women only, the remainder being for both sexes.

---

### POLITICS AND BILIOUSNESS.

*Le Figaro* publishes a most humorous article entitled "M. Challemel-Lacour's Liver," demonstrating that the destinies of the nation are at the mercy of a bilious man who signs treaties or threatens war according to the state of his liver at the moment he is discussing affairs with the representative of a foreign power.

